# CT scan evaluation of glenoid bone and pectoralis major tendon: interest in shoulder prosthesis

**DOI:** 10.1051/sicotj/2016021

**Published:** 2016-10-07

**Authors:** Laurent Obert, Christelle Peyron, Etienne Boyer, Gauthier Menu, François Loisel, Sébastien Aubry

**Affiliations:** 1 Orthopaedic and Traumatology Surgery Service, University Hospital of Besançon 25000 Besançon France; 2 Intervention, Innovation, Imagery, Engineering in Health (EA 4268), Medical and Pharmacology Section, IFR 133, University of Franche-Comté 25000 Besançon France; 3 Radiology unit, Research Unit: EA 4268 I4S - IFR 133 INSERM University Hospital of Besançon 25000 Besançon France

**Keywords:** Glenoid, Pectoralis major muscle, Shoulder prothesis, Hemiarthroplasty, Proximal humerus fracture

## Abstract

*Introduction*: The shoulder arthroplasty brings satisfaction to patients in terms of quality of life and indolence. However whether anatomic implant or reverse, it does not escape from the loosening of the glenoid component. Moreover, optimal implantation is required to ensure the functional outcome without shortening of the arm. The purpose of this study is obtain CT scan evaluation of the glenoid bone stock in order to optimize glenoid component implantation and obtain a reference to determine optimal humeral component placement in case of humeral proximal fracture.

*Materials and methods*: Between 2010 and 2011 we have analyzed 200 intact shoulder’s CT. We measured maximal and minimal width in the transverse plane of the glenoid, the distance from the pectoralis major (PM) tendon to the humeral head, the greater tubercle, change of curvature and the anatomical neck.

*Results*: Mean maximum width was 27.4 ± 3.4 mm and mean minimum width was 15.5 ± 2.8 mm. Distances between upper edge of PM tendon to: humeral head, greater tubercle, change of curvature and anatomical neck were respectively: 67.6 ± 9.98 mm, 57.8 ± 10.3 mm, 28.7 ± 9 mm, and 34.2 ± 9.7 mm.

*Conclusion*: Our study has produced an assessment of glenoid bone stock for optimal positioning of the glenoid implant but also to obtain a reference to determine the ideal location of the humeral component in the case of proximal humerus fracture.

## Introduction

Gluck, born in 1853 in Jassy, Romania, was the first surgical pioneer to record a shoulder replacement, performed with the use of an ivory prosthesis. In 1893 Jules Emile Pean, a French surgeon, implanted a prosthesis to replace a glenohumeral joint that had been destroyed by tuberculosis [1]. Very little progress was made until 1951 when Charles Neer developed a prosthesis for proximal humerus. Influenced by the success of total hip arthroplasty, Neer developed the first total shoulder prosthesis, the Neer II (1974). It consisted of a humeral component and a glenoid resurfacing component [2]. In 1985, Paul Grammont developed a new concept; the reverse total shoulder arthroplasty for the treatment of a severely degenerated glenohumeral joint with nonreconstructible rotator cuff rupture [3, 4]. Even if patients are satisfied with shoulder prosthesis [5, 6] in terms of quality of life, functional outcomes, and benignity [4, 7], it does not appear to be a long-lasting solution. Some of the common causes of failure in reverse total shoulder arthroplasty involve loosening of the glenoid component. It is the most common complication (39%) [8] causing a resumption of pain, limitation of function and, potentially, needing for revision surgery [9]. Thus, the weak structure of total shoulder prosthesis (anatomic or reverse), used in osteoarthritis, is glenoid component fixation. Humeral component, only, is the treatment of proximal humeral fracture. Optimal implantation is mandatory to assure functional outcome without shortening of the arm. There were previous anatomic and radiologic studies to understanding shoulder biomechanical and optimal implant positioning. Nevertheless, these studies [3, 4, 10–30] were performed from only several specimens. These results must be confirmed on a larger sample. Moreover, these studies do not examine the relationship between bone and Pectoralis Major (PM) tendon which is an essential marker, on pre-operative, for optimal implantation of humeral component.

Thus, the purpose of this study is to get CT scan evaluation of glenoid bone stock in order to optimize glenoid component implantation and get a reference to determine optimal humeral component placement in case of humeral proximal fracture.

Secondary aims are to determine the influence of gender and osteoarthritis on these results.

## Materials and methods

Two hundred intact shoulder CT scans acquired between 2010 and 2011 were analyzed. Our population included 88 women and 112 men with a mean age of 49.8 years ± 18.5 (from 18 to 93 years old); 17.5% showed osteoarthritic lesions (35/200), 28.5% showed a rupture of the cuff (57/200). All scans were analyzed by the same observer with multiplanar reconstructions on posttreatment consol PACS. Imaging parameters were as follows: helical scan type, 64 detector rows, 140 KeV, 350 mA, one second rotation time, 1 mm helical thickness, pitch 0.8.

**Figure 1.**
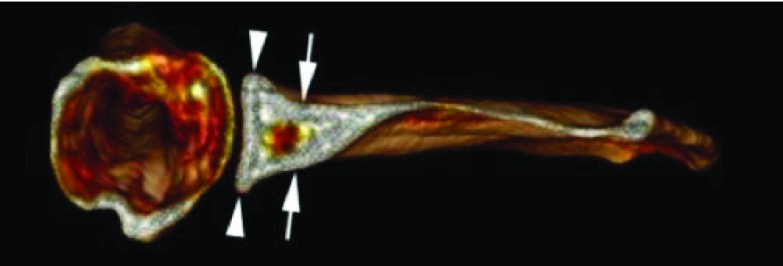
Maximal width (arrowhead) and minimum width (arrow).

**Figure 2.**
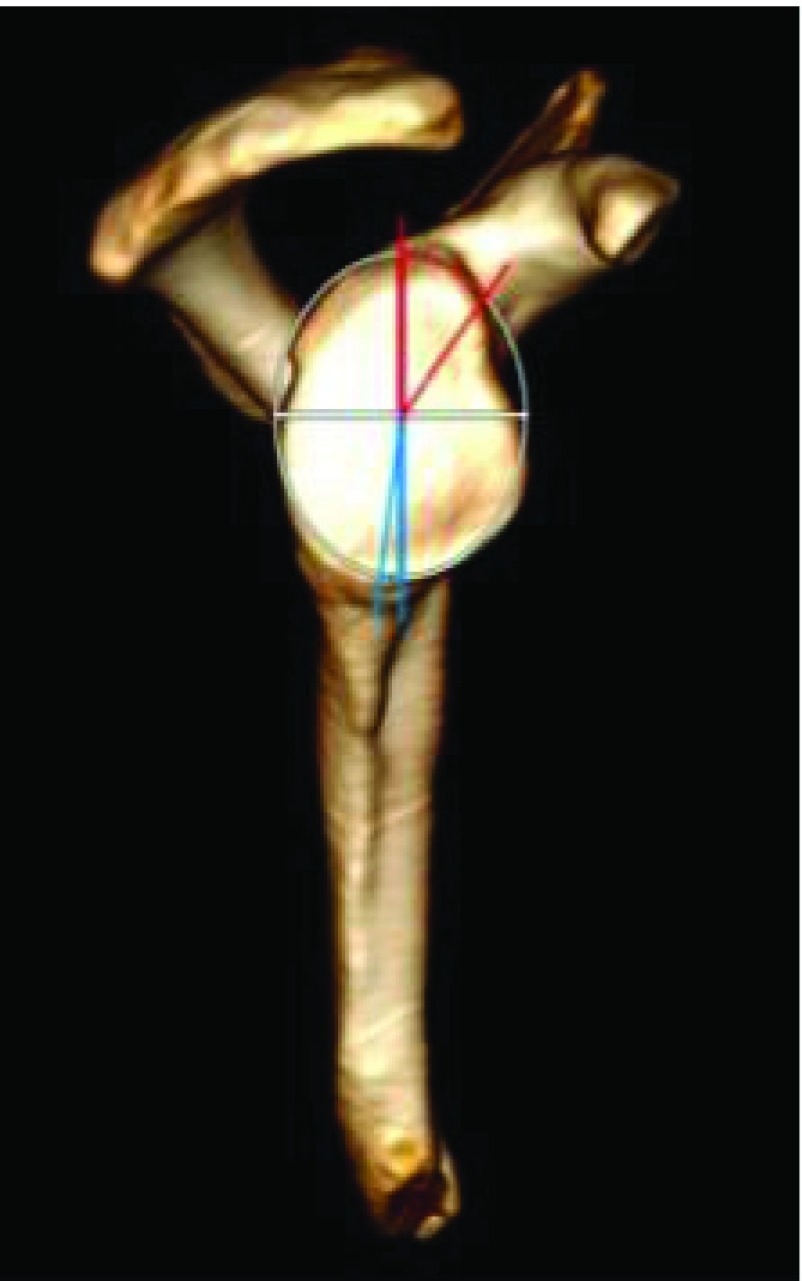
Higher angle (red) and lower angle (blue).

**Figure 3.**
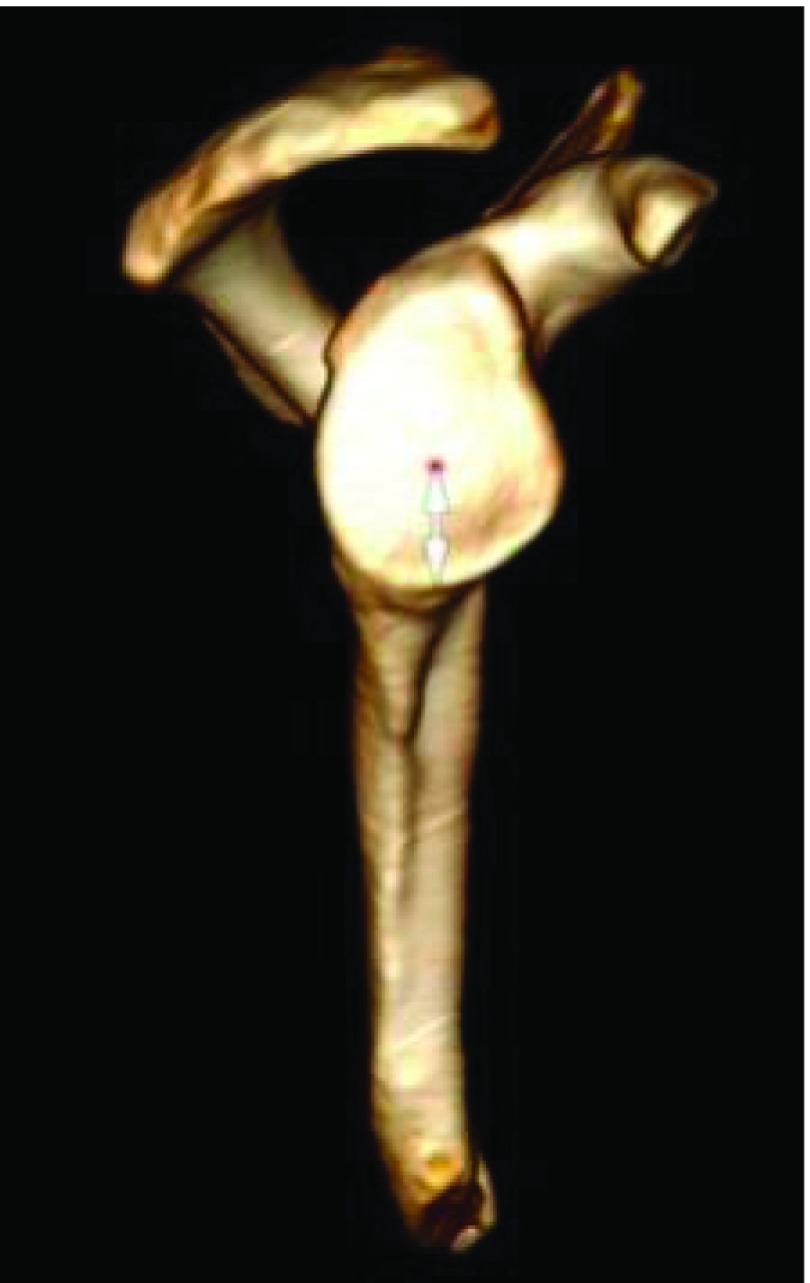
“12 mm point”.

Various morphologic parameters were measured on the glenoid, from each CT scan: maximal width in transverse plane and minimum width next to the base of coracoid process in transverse plane (Figure 1), higher angle (defined between North-South axis and the spine) and lower angle (defined between North-South axis and upper posterior column of the scapula) (Figure 2), and the “12 mm point”: the distance from maximal bone stock to inferior glenoid rim (Figure 3).

**Figure 4.**
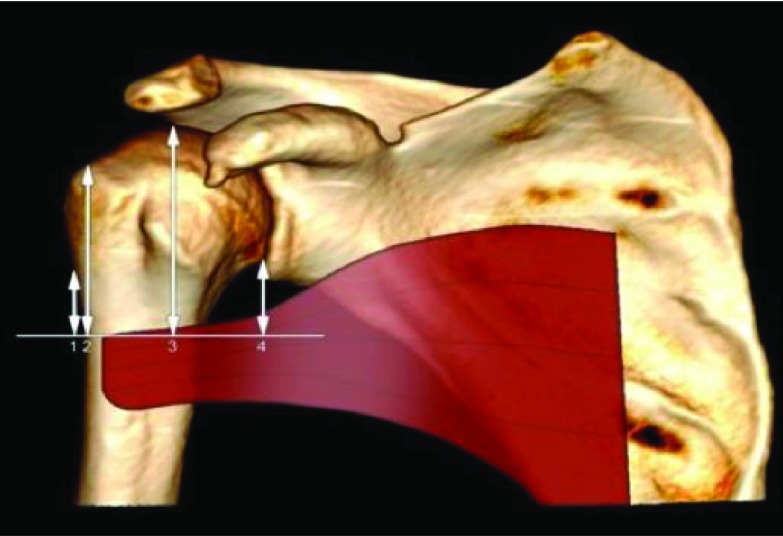
Distance between the upper edge of PM tendon to change of curvature (1), great tubercle (2), humeral head (3), and anatomical neck.

In 137 CT scans where the PM was identifiable we measured the distance between the tendon to humeral head, greater tubercle, change of curvature, and anatomical neck (Figure 4). The PM muscle was identified in axial, in “soft tissue” window and followed up to its insertion on the humerus.

## Results

### Glenoid dimensions

**Figure 5.**
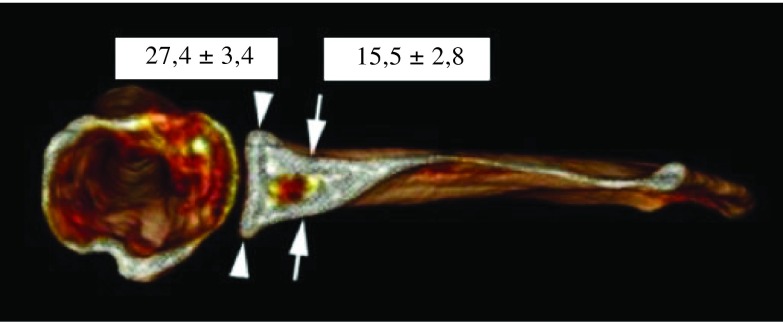
Mean values ± SDs for maximum and minimum width.

**Figure 6.**
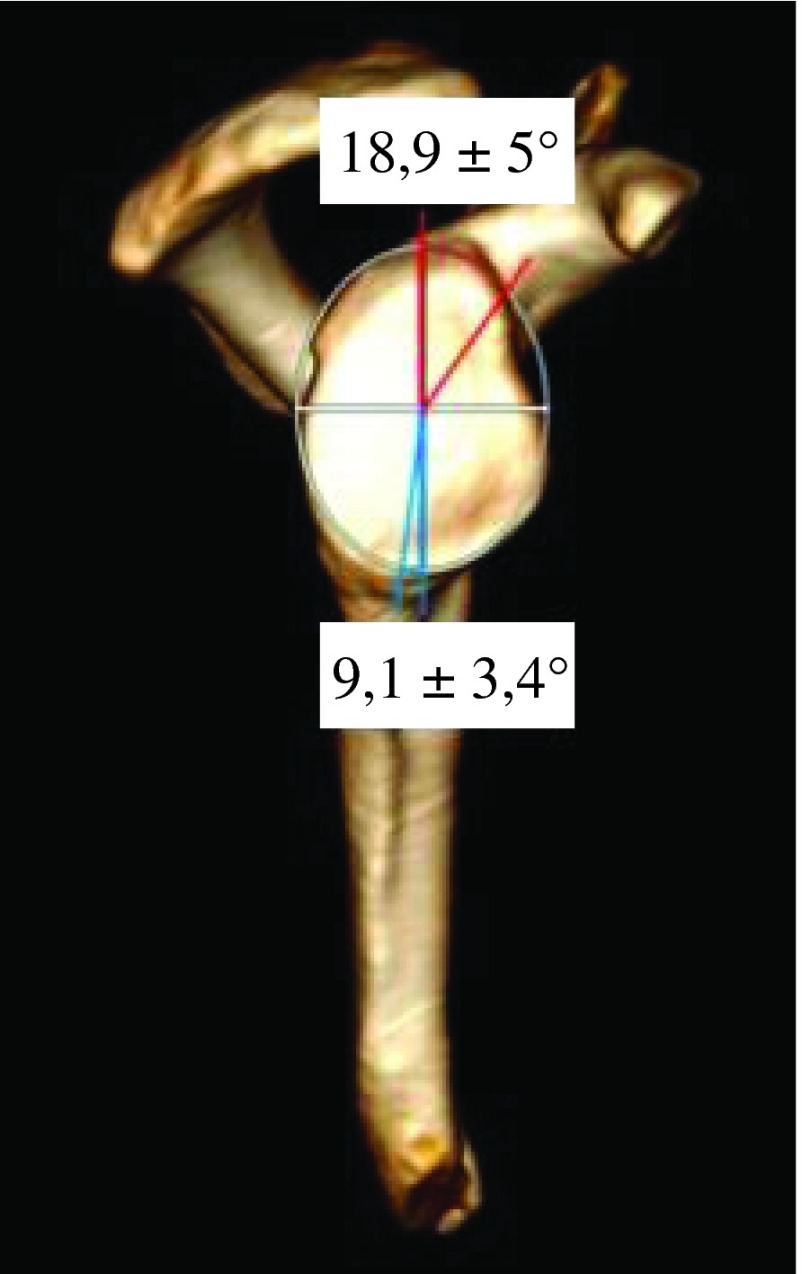
Mean values ± SDs for higher angle and lower angle.

**Figure 7.**
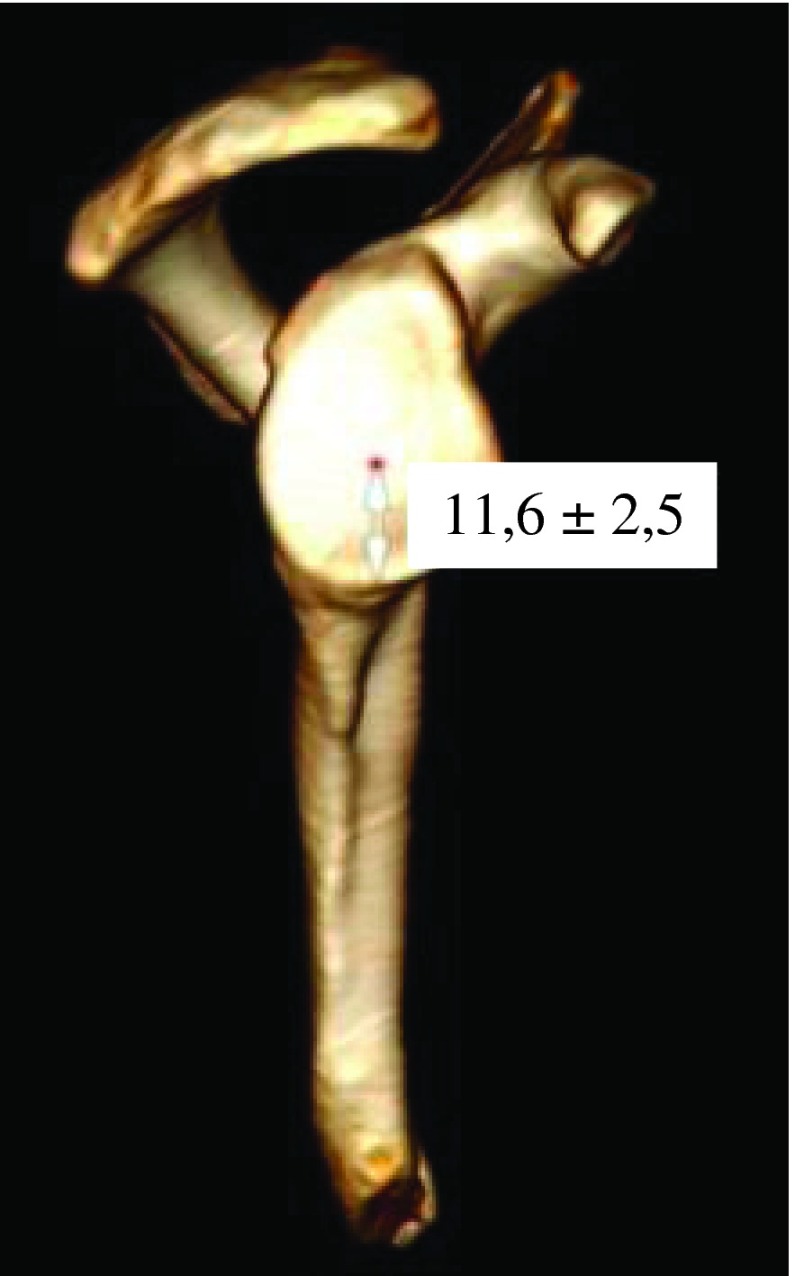
Maximal bone stock.

Mean maximum width was 27.4 ± 3.4 mm (from 18 mm to 39 mm). Mean minimum width was 15.5 ± 2.8 mm (from 9 mm to 22 mm) (Figure 5). Mean maximum width on mean minimum width was 1.8 ± 0.3. For woman mean maximum width was 25.4 ± 3.5 mm, while for men, it was 28.9 ± 2.4 mm. Mean maximum width was higher for males (3.5 mm, *p* < 0.001). Maximum width did not demonstrate any relationship with age (*p* = 0.073). In case of osteoarthritic lesion, mean maximum width was 28.8 ± 3.8 mm. Intact scapulae’s mean maximum width was 27.1 ± 3.2 mm. Maximum width was significantly higher in case of osteoarthritis (1.7 mm higher *p* = 0.017). Mean higher angle was 18.9 ± 5° and mean lower angle was 9.1 ± 3.4° (Figure 6). Maximal bone stock was at 11.6 ± 2.5 mm above inferior glenoid rim (from 6 mm to 19 mm) (Figure 7).

### Humeral height

**Figure 8.**
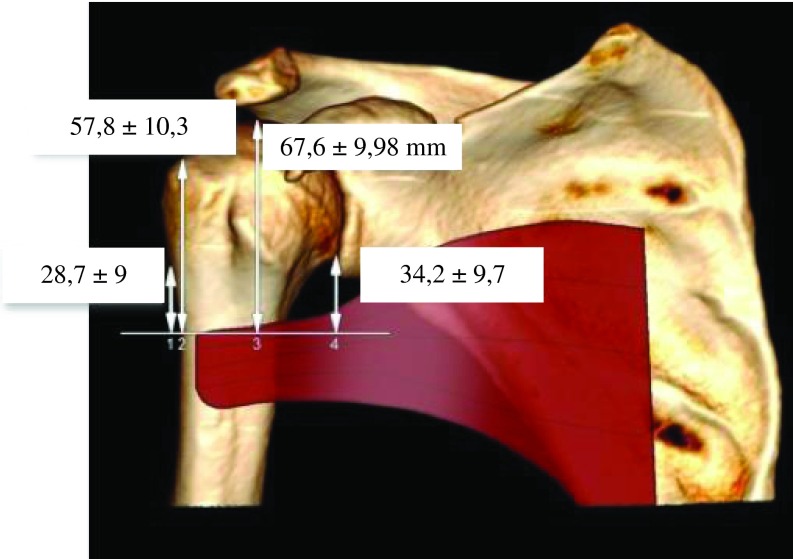
Mean values ± SDs for humeral height.

Distances between upper edge of PM tendon to: humeral head, greater tubercle, change of curvature, and anatomical neck were, respectively: 67.6 ± 9.98 mm (from 48 mm to 94 mm), 57.8 ± 10.3 mm (from 39 mm to 84 mm), 28.7 ± 9 mm (from 11 mm to 57 mm), and 34.2 ± 9.7 mm (from 9 mm to 57 mm) (Figure 8). The PM-humeral head distance was 62.95 ± 7.9 mm for women and 70.7 ± 10 mm for men. There was a significant relationship between PM-humeral head distance with gender: 7.8 mm higher for male patients (*p* < 0.001). The PM-greater tubercle distance was 53.3 ± 7.8 mm for women, 60.9 ± 10.7 mm for men. There was a significant relationship between PM-greater tubercle distance with gender: 7.6 mm higher for male patients (*p* < 0.001). Moreover, there was a significant relationship between maximum width and PM-greater tubercle distance (*p* = 0.003).

We have made ratios between the different values of humeral height. Their results are presented in Table 1.

**Table 1.** Humeral height ratios.

Ratios	Results
PM-change of curvature with PM-anatomical neck	0.9 ± 0.2
PM-greater tubercle with PM-change of curvature	0.5 ± 0.1
PM-anatomical neck with PM-great tubercle	0.6 ± 0.1
PM-humeral head with PM-greater tubercle	0.9 ± 0.1
PM-humeral head with PM-change of curvature	0.4 ± 0.1
PM-humeral head with PM-anatomical neck	0.5 ± 0.1

**Figure 9.**
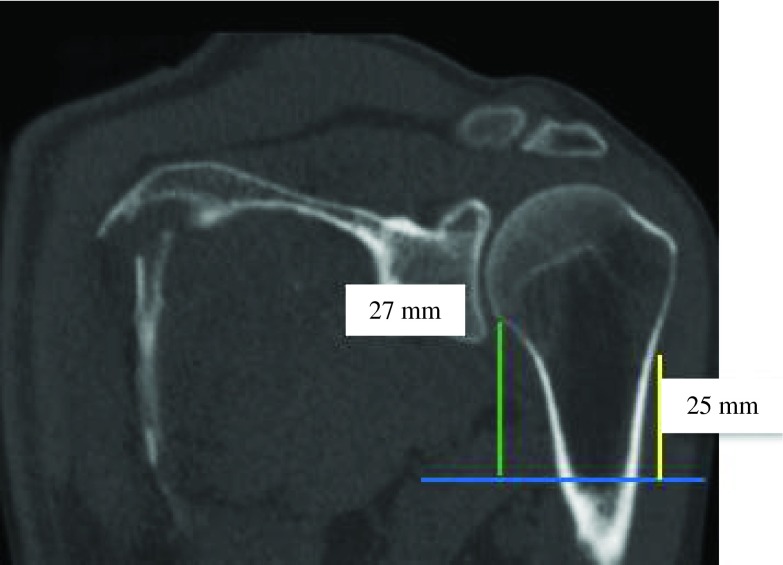
PM-change of curvature distance (yellow) was almost equal to PM-anatomical neck distance (green) (ratio = 0.9).

**Figure 10.**
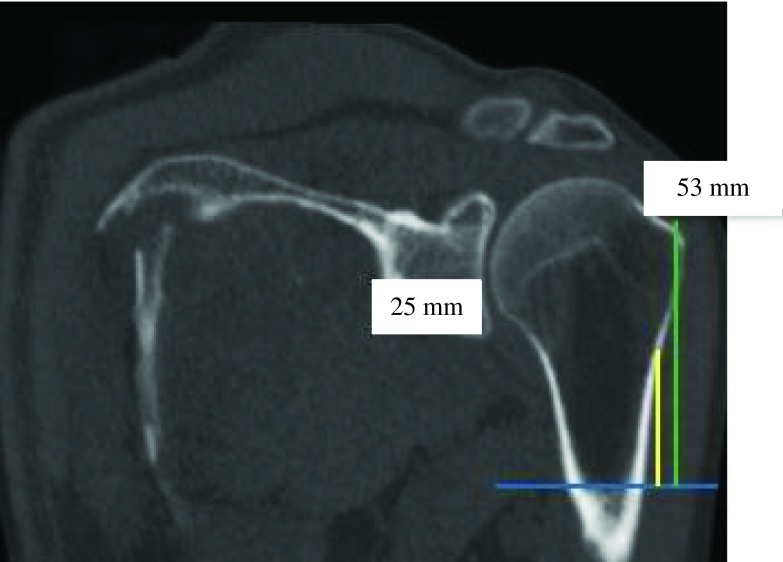
PM-change greater tubercle distance (green) was two times greater than PM-change of curvature distance (yellow) (ratio = 0.5).

**Figure 11.**
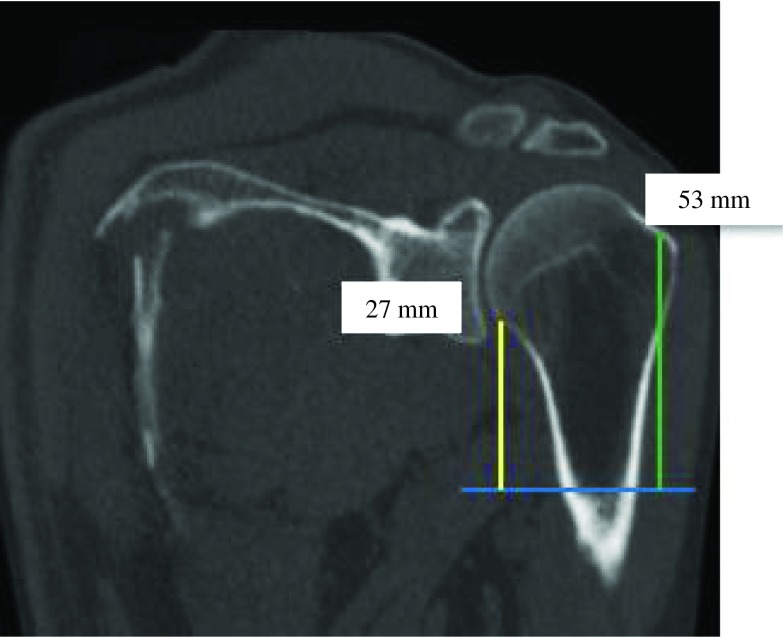
PM-greater tubercle distance (green) was almost equal to PM-anatomical neck distance (yellow) (ratio = 0.6).

**Figure 12.**
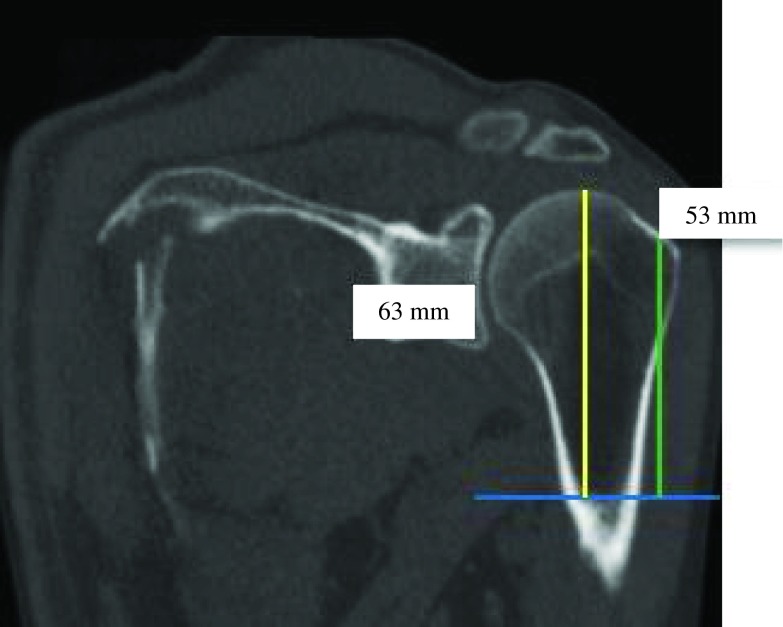
PM-humeral head distance (yellow) was almost equal to PM-greater tubercle distance (green) (ratio = 0.9).

**Figure 13.**
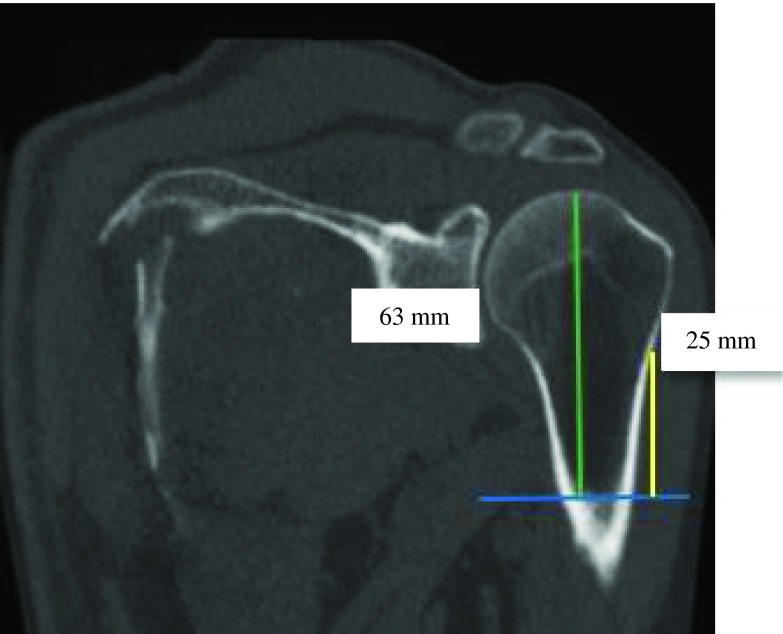
PM-humeral head distance (green) was almost two times greater than PM-change of curvature distance (yellow) (ratio = 0.4).

**Figure 14.**
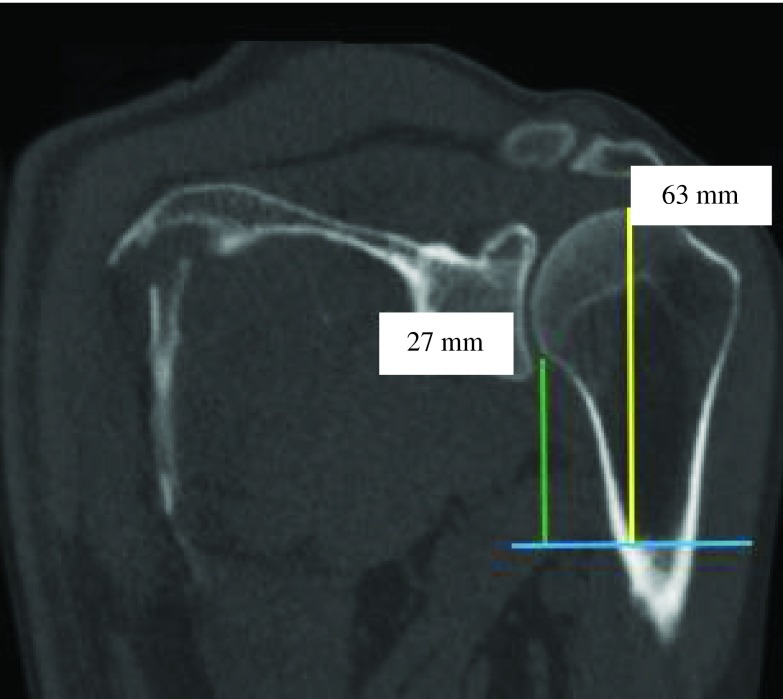
PM-humeral head distance (yellow) was two times greater than PM-anatomical neck distance (green) (ratio = 0.5).

PM-anatomical neck and PM-great tubercle distances were equal like PM-great tubercle and PM-humeral head distances. PM-great tubercle and PM-humeral head distances were two times greater than PM-change of curvature and PM-anatomical neck distances. There was a significant relationship between age and osteoarthritis. The mean age of patients with intact shoulder was 46.6 ± 17.5 years. Patients with osteoarthritis were older (64.8 ± 15.8 years) (*p* < 0.001) (Figures 9–14).

## Discussion

### Glenoid measurements

There were many studies whose purpose was to describe glenoid anatomy [11, 13, 15, 19, 24, 31–38].

Most of these studies were realized from few specimens. For example, Hoenecke et al. in 2008 [32] used 40 cadavers. He showed mean maximum width at 27.9 mm. He showed osteoarthritic lesion caused glenoid deformation that the surgeon must take into account. The changes of glenoid shape caused by osteoarthritis have been shown by Codsi et al. [13] too, from 61 skeletal specimens. Our glenoid morphometric measurements were consistent with those from previous studies realized from many specimens. Franckle et al. [19], from 216 scapulae, found that maximum width at 28.9 ± 3.3 mm. Churchill et al. [33], from 344 specimens, found a maximum width ranged from 23.4 mm to 28.1 mm. Checroun et al. [31], from 412 specimens, showed a maximum width of 29.3 ± 1.5 mm. Checroun et al. and Churchill et al. [31, 33] found a significant relationship between mean maximum width and gender, like our study.

All the results are summarized in Table 2.

**Table 2.** Results of mean maximum width (mm).

Series	Our study	Franckle	Checroun	Churchill
Sample	200	216	412	344
Maximum width	27.4 ± 3.4	28.9 ± 3.3	29.3 ± 2.4	27.8 ± 1.6
Men	28.9 ± 2.4	/	/	27.8 ± 1.6
Women	25.4 ± 3.5			23.6 ± 1.5

The choice of glenoid component must be adapted to gender. Thus, the male component must be larger than the female component. We could propose a 29 mm for male patients and a 25 mm for female patients. The glenoid implants available on the market are bigger than our anatomical results. Peripheral screw placement is essential for glenoid component stability [14, 39]. There were few anatomic studies that described bone stock localization in coracoid process and in column [14, 17, 22, 28, 36]. Humphrey [22] developed his three column concept about 10 skeletal specimens. He showed higher angle at 19° and lower angle at 14°. Torrens et al. [28], about 108 specimens, found a higher angle ranged from 13° to 27° (average = 18.3°) and lower angle ranged from 5° to 180 (average = 8°). Our study was consistent with Humprey because we showed mean higher angle at 18.9° and mean lower angle at 9°, ranged from 7° to 34° and from 3° to 21°. Angle measurements showed a high variation according to glenoid shape. If glenoid is elongated, higher angle is acute. Contrary superior angle is bigger if glenoid is pear shaped. To reduce scapular notching, glenoid component must be lowered. Nevertheless, the central keel must be implanted where bone stock is maximal. There was one previous study [3], about 10 specimens that described 12 mm between glenoid inferior rim and maximal bone stock. Our study confirmed its results, from a larger number of patients.

### Humeral height

Humeral height measures are useful for the surgeon to position prosthesis and get successful outcome after humeral arthoplasty for humeral head fracture. There were several cadaveric studies that showed PM tendon like a reference. But, these studies used 40 specimens maximum [21, 25, 29]. Thus, Murachovsky et al. [25] showed from 40 cadavers (11 male patients) a PM-humeral head distance ranged from 50 to 70 mm (average = 56.4 ± 5 mm). Torrens et al. [29], in 2008, found from 20 specimens (12 men and 8 women), a PM-humeral head distance ranged from 53 mm to 60 mm (average = 56.4 mm). Hasan et al. [21] found a PM-humeral head distance at 57.7 mm ± 6.1 mm. All these studies are cadaveric studies while our study was performed on living people. All the results are summarized in Table 3.

**Table 3.** Results of humeral height (mm).

Series	Our study	Murachowsky	Torrens	Hasan
Sample	137	40	20	38
PM-Humeral head	67 ± 10	56 ± 5	56.4 ± 5	57.7 ± 6.1
Variations	48–94	50–70	53–60	46.6–71.6

Our study showed a PM-humeral head distance at 67 ± 10 mm higher than others studies. This difference could be explained because our workforce was higher with more male patients. We showed a significant relationship between PM-humeral head distance with gender (this distance was 7.8 mm higher in males patients). Another time, surgeon must take care of gender and must adapt component implantation according to gender and stature. Nevertheless, this measure can be realized by an anterior approach [40]. By superior approach, another landmark is useful like PM-anatomical neck distance. Thus, we showed PM-anatomical neck distance at 34 ± 9.37 mm two times smaller than PM-humeral head distance. At last, the measurement of PM-change of curvature could vary depending on the surface anatomy of proximal humerus but in fact it did not change significantly the results.

Optimal component positioning is essential to get useful outcome. Thus, too low implantation leads to too much tension on deltoid muscle and leads to secondary fracture of acromion. On the contrary, too high implantation leads to not enough tension and leads to bad clinical outcome. Renaud et al. [4] showed that with optimal positioning, rehabilitation is fairly short and easy. Thus, outcomes concerning pain and functional result were good. Nevertheless, he used acromio-epiphyseal distance not PM-humeral head distance. Pre-operative evaluation for a total shoulder arthroplasty includes standard radiographs [40]. CT scans with 3D reconstructions, MIP analyses are more useful. 3D reconstructions of CT images allow 3D visualization and analysis of the scapula and can reflect the true anatomy [35] and provide valuable information regarding the glenoid vault. 3D CT images are a useful tool during the pre-operative evaluation for a total shoulder arthroplasty, particularly in patients with significant glenoid bone loss. There were several limitations of this study. First, PM tendon cannot be always identifiable certainly. So, we obtained humeral length from 137 CT scans only. In the same way, angle measurements were difficult to be realized precisely because of osteoarthritic lesions, which changed glenoid shape.

## Conclusion

Our study was the first in vivo, on a large number of patients. It showed maximal bone stock and provided new data useful for a good fixation of shoulder prosthesis. Our results were consistent with results from previous anatomic studies. We showed glenoid width depends on gender. In the same way, we showed that component’s implantation and component choice must depend on the gender and stature of the patient.

## Conflict of interest

The authors declare no conflict of interest in relation with this paper.
